# Annotations

**Published:** 1891-12-12

**Authors:** 


### ANNOTATIONS.

**Medicine and Surgery at Toronto.**

NOTHING better shows the vitality and activity of the Medical science of the times than the fact that wherever it is possible what are called Post Graduate courses of lectures in medicine and surgery are established, and are attended by large numbers of working practitioners. Doctors feel, what all sorts and conditions of men ought to feel, that a mental attitude of life-long discipleship is the only proper attitude for any intelligent man to occupy in such a world as ours. That medicine, which was for so many centuries a bye-word and a scandal to the scientific, should now lead the way in so truly scientific a temper is highly honourable to the physicians and surgeons of the present time. The University of Toronto, which is but a "baby" University compared with many of our European seats of learning, is not a whit behind the most advanced and enthusiastic of them all in respect of this Post Graduate work. We have been favoured with a published copy of nine Post Graduate lectures on medical and surgical subjects of the highest scientific and practical importance by nine medical professors, chiefly of the Toronto University. The lectures, as a whole, are not only worthy of the professors of the University, but of any body of professors in any part of the world. We do not say that everything which is advanced is demonstrated, or that there are no traces whatever of crude theorising and hasty generalisation. But we say that there is no more evidence of such lack of perfect culture in these lectures than there would be in the lectures of any nine medical professors taken from almost any University in the world. The first lecture is one by Professor White on "The Present Position of Antiseptic Surgery." Professor White is an ardent Listerian, and, we think, is, on the whole, justified in holding the position he occupies. In defending Sir Joseph Lister he hits out very boldly at Mr. Lawson Tait. He hardly does justice, we think, to Mr. Tait. "Get out all the decomposable matter from any given wound or cavity," says Mr. Tait, "and you can let in the germs freely." Professor White would be the last to attempt to deny that there is a very great deal of truth in this proposition. Moreover, Mr. Lawson Tait's almost unparalleled success in operations of an admittedly dangerous kind, without active antiseptics, is a strong justification of his position from his own point of view. Professor White also does somewhat less than justice to the late Professor Spence. The present writer followed the practice of both Spence and Lister in their wards at Edinburgh, and saw the results of their work. Spence was an admirable and a successful surgeon. He believed in asepsis if he did not believe in antiseptics. Lister had the merit and the advantage of believing in both the one and the other. Certainly Lister had some remarkable triumphs at Edinburgh. It was a sight never to be forgotten to see him holding up dressings which had been put on newly-closed wounds, and had been left on for several days. The way in which he himself smelt them first, and then handed them round for the students to smell, was quite dramatic. No student who followed Lister in those days and heard his lectures failed to become a convinced antisepticist. The writer is still a believer in the antiseptic treatment. But he also believes in the aseptic. If you can make your asepsis perfect, your antisepsis becomes at once superfluous. But you can never depend upon making your asepsis perfect, and that is where Mr. Lawson Tait fails to do justice both to Sir Joseph Lister and to himself. Asepsis, combined with antisepsis, is the true and philosophical position to take up. The controversy between Sir Joseph Lister and Mr. Lawson Tait, carried on mainly by the latter, is inconsequent and foolish. It is unworthy of Mr. Tait. He apparently cannot forgive Sir Joseph Lister for having neglected him. Sir Joseph's fame is fixed on a secure and permanent basis. He had the rare merit of bringing in antisepsis by his own philosophic work and scientific enthusiasm. Asepsis followed as an inevitable corollary. The two together constitute the last word on the subject. Sir Joseph Lister knows this, and Mr. Lawson Tait, at least, ought to know it.

**The Carpenters' Company and the Sanitary Institute.**

All persons who recognise the dangers of crowding together vast populations in single towns may congratulate themselves when public bodies begin in earnest to strive to make the conditions of town life as healthy as possible. The ancient Egyptians were practical sanitarians; the Europeans of the middle ages were practically fools in sanitary matters. The narrow streets and the miserably inadequate domestic and public drainage of most European and English towns that date several centuries back, constitute a standing disgrace to civilisation. People have been content with houses, drains, streets, and all sorts of sanitary appliances of the most haphazard and ridiculous kind. The results have been more destructive to life and property than all the wars in which Europe ever engaged. We are beginning to do better, but only beginning. We attack a little problem here and there. What we ought to do should be to equip a sanitary standing army, to pay it well, and to give it powers to deal effectually with all our poisons, and rotten drains, and dwellings. However, if half a loaf is better than no bread, we may be thankful that beginnings are being made here and there. What we wish to call attention to, at the present moment, is the fact that the Worshipful Company of Carpenters has joined hands with the Sanitary Institute for the purpose of establishing "Examinations" in practical sanitation, and in building, so far as it relates to sanitary construction. The examinations will be held at convenient times and places; and certificates will be awarded to those ex-candidates who reach a reasonable standard of proficiency. Such certificates, awarded by such a board of examiners, will be of very great value both to the classof working builders and to their employers. They will, of themselves, be almost sufficient proof to masters, that particular workmen are fit and proper persons to be employed as foremen. Moreover, such a certificate held by a master builder will be an assurance to the public that he at least possesses a fair amount of rational knowledge of practical sanitation and building. The examination will include such subjects as a general knowledge of materials used in building; the properties and qualities of bricks, stone, lime, and cement; methods of making and using mortar, cement, and concrete; also the nature and properties of timber, its decay and preservation. It will further deal with sites of buildings, air spaces, construction of foundations, of walls, floors, and roofs, with sanitary arrangements, including water-closets, privies, and dry closets, water supply, tanks and services, and plumber's work. It will include also every part of drainage, ventilation, warming, and other technical details. Moreover, a special knowledge of the Acts of Parliament relating to buildings and their sanitation, issued by the Local Government Board, will be required. All this is eminently satisfactory. The Board of Examiners is one which will command the respect and confidence of all who understand the subject. We would venture to point out by way of friendly criticism that the examination fee of three guineas, which is demanded, is considerable, and ought to be subject to modification. A master builder or his son may very properly pay such a fee. But a youth newly out of his apprenticeship may find it inconvenient to spare so much. One guinea would be a more fitting fee for him to pay. Then all candidates who fail to satisfy the Examiners the first time are asked to pay half the fee for a second examination. This we think is neither just nor wise, especially if the three guinea fee be maintained for all classes of candidates. A second examination should certainly be granted without additional fee to a man who has paid his full fee for the first. If a third trial be needed by any particular candidate, then he may properly be asked to pay half the original fee. These are details, but not unimportant ones; and we hope the Committee will see their way to a reconsideration of the subject. We congratulate both them and the carpenters and builders, as well as the general public, on the improved sanitary prospect that lies before the world of Englishmen.

